# Brown tumor of the cervical spine with primary hyperparathyroidism: A case report and literature review

**DOI:** 10.1097/MD.0000000000032768

**Published:** 2023-02-10

**Authors:** Zirui Liu, Hao Yang, Hongyu Tan, Ruipeng Song, Yang Zhang, Liang Zhao

**Affiliations:** a Department of Orthopaedics, The First Affiliated Hospital of Zhengzhou University, Zhengzhou, China.

**Keywords:** brown tumor, primary hyperparathyroidism, spine

## Abstract

Brown tumor (BT), an uncommon focal lytic bone tumor, is a non-neoplastic and reactive process caused by increased osteoclastic activity and fibroblastic proliferation in primary or secondary hyperparathyroidism. Vertebral tumor causing neural compression is relatively rare, especially in the cervical spine.

**Patient concerns:**

A 29-year-old man developed neck pain and arm radicular pain 4 months ago, with the level of serum calcium significantly higher than normal. Computed tomography scan of the cervical spine revealed an expansile lytic lesion occupying the C6 body, left pedicle, and left lamina of C5–6.

**Diagnoses:**

Osteoclastoma according to imaging and histopathological results.

**Interventions:**

A laminectomy of C5–6 was performed.

**Outcomes:**

One month later, he was re-hospitalized due to nausea and vomiting and the serum calcium, was still, kept at a high level. Additionally, the parathormone (PTH) was greatly higher than normal. BT with primary hyperparathyroidism due to the parathyroid tumor was considered. After the surgery of the right parathyroid gland was performed, serum calcium and PTH both decreased, and computed tomography showed good recovery.

**Lessons:**

BTs might be misdiagnosed as other giant cell tumors, thus when giant cell tumors are considered, serum calcium and PTH examination may be needed to exclude BTs.

## 1. Introduction

Brown tumor (BT) is a rare benign focal lytic bone lesion that arises in the context of primary or secondary hyperparathyroidism.^[1]^ It was found in <5%^[2]^ of patients with primary hyperparathyroidism while the incidence in secondary hyperparathyroidism is 1.5% to 13%.^[3,4]^ BT can appear as solitary or multiple lesions of any bone and mainly involve extremities, sternum, clavicle, ribs, mandible, and pelvis.^[5–7]^ Involvement of the spine is unusual, and cervical and multiple spine involvements are extremely rare. Due to it being rare, BT can be misdiagnosed with more malignancy spine lesions. Here we described a BT that was initially mistaken for osteoclastoma. To our best knowledge, this is the first male and cervical case of BT and multiple BT due to primary hyperparathyroidism. And the present report emphasized the importance of distinguishing BTs from other giant cell tumors of the bone and the relevance of measuring serum calcium and parathormone (PTH) before the diagnosis of osteolytic bone lesions. This can significantly impact the correct diagnosis and escape unnecessary surgery.

## 2. Case report

A 29-year-old man was hospitalized with a 4-month history of neck pain and radicular pain in both upper limbs. He had enjoyed good health until 4 months prior to this admission, when he began to suffer from progressive neck and arms pain, mostly on the left side. The pain was exacerbated by fatigue and movement and was eased during bed rest. On physical examination, the cervical muscles were very tender to palpation. On neurological examination, tendon reflexes were normal and symmetrical.

The fasting serum calcium varied between 2.98 and 3.51 mmol/L (normal 2–2.7 mmol/L), and the following blood chemistry was found: blood urea 4.30 mmol/L, serum creatinine 70 μmol/L, serum uric acid 438 μmol/L, glomerular filtration rate 120.38 mL/min/1.73 m^2^, and the electrocardiogram showed a shortened Q-T interval consistent with hypercalcemia.

**Figure 1.**
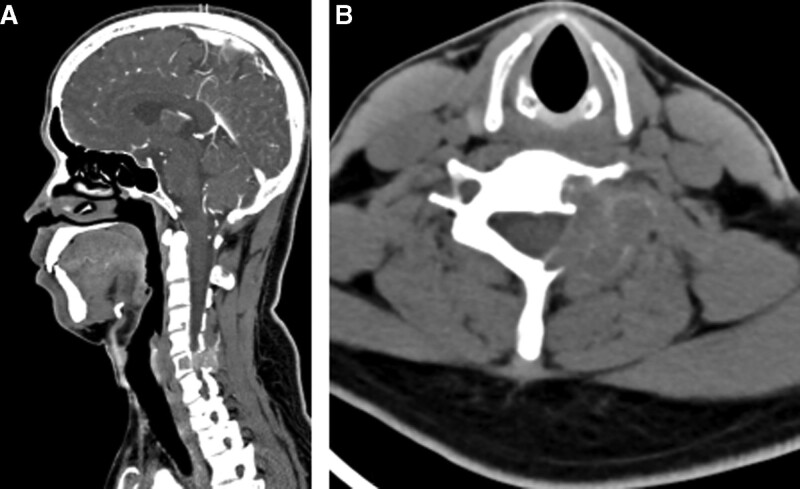
Sagittal (A) and axial (B) CT images reveal a hyperdensity expansile lesion at C6 level.

**Figure 2.**
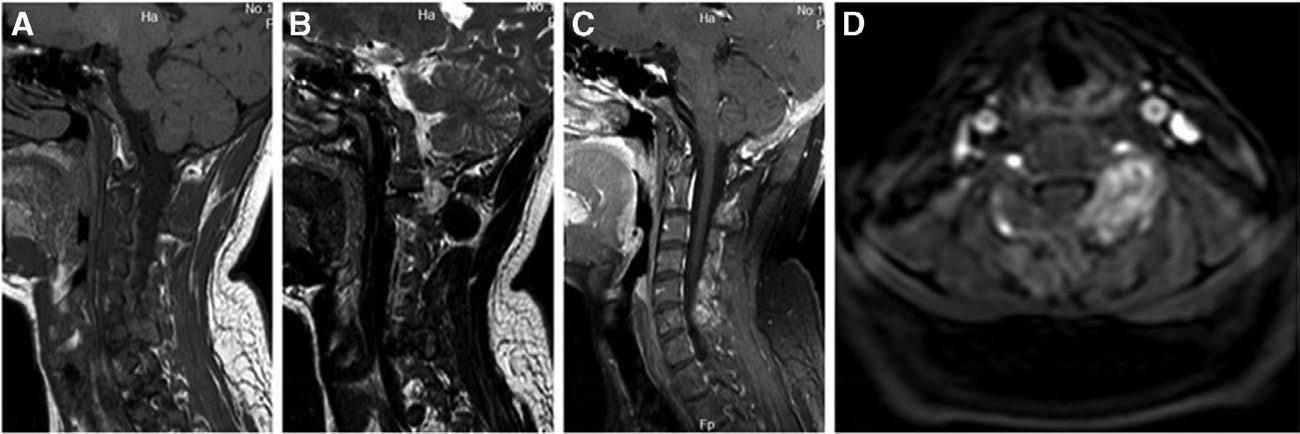
Sagittal T1W (A) MRI shows an isointense lesion, sagittal T2W (B) MRI, sagittal demonstrates a hypointense lesion, T1W postcontrast (C) MRI reveals a homogenously enhancing expansile lesion and axial T2W (D) MRI image shows the lesion encase the left vertebral artery at C6 level. MRI = magnetic resonance imaging.

X-ray showed a normal cervical vertebral. Computed tomography (CT) scan of the cervical spine revealed an expansile lytic lesion occupying the C6 body, left pedicle, and the left lamina of C5-6 (Fig. 1). Magnetic resonance imaging (MRI) with contrast showed the lesion occupying the body of C6, and the left vertebral artery was encased with paravertebral soft tissue mass. The lesion showed hypointense on T2-weighted images and isointense on T1-weighted images (Fig. 2). Provided these imaging features, osteoclastoma, and the aneurysmal bone cyst should be considered.

**Figure 3.**
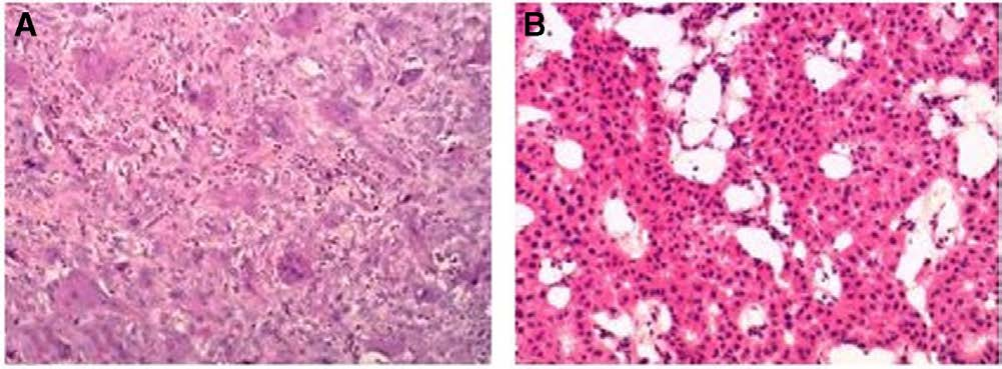
Histopathological examination of the lesion (A) shows clusters of giant cells and hemosiderin deposition (hematoxylin & eosin × 100). Histopathological examination of the parathyroid (B) demonstrates an adenoma consisting mostly of chief cell type (hematoxylin & eosin × 100).

**Figure 4.**
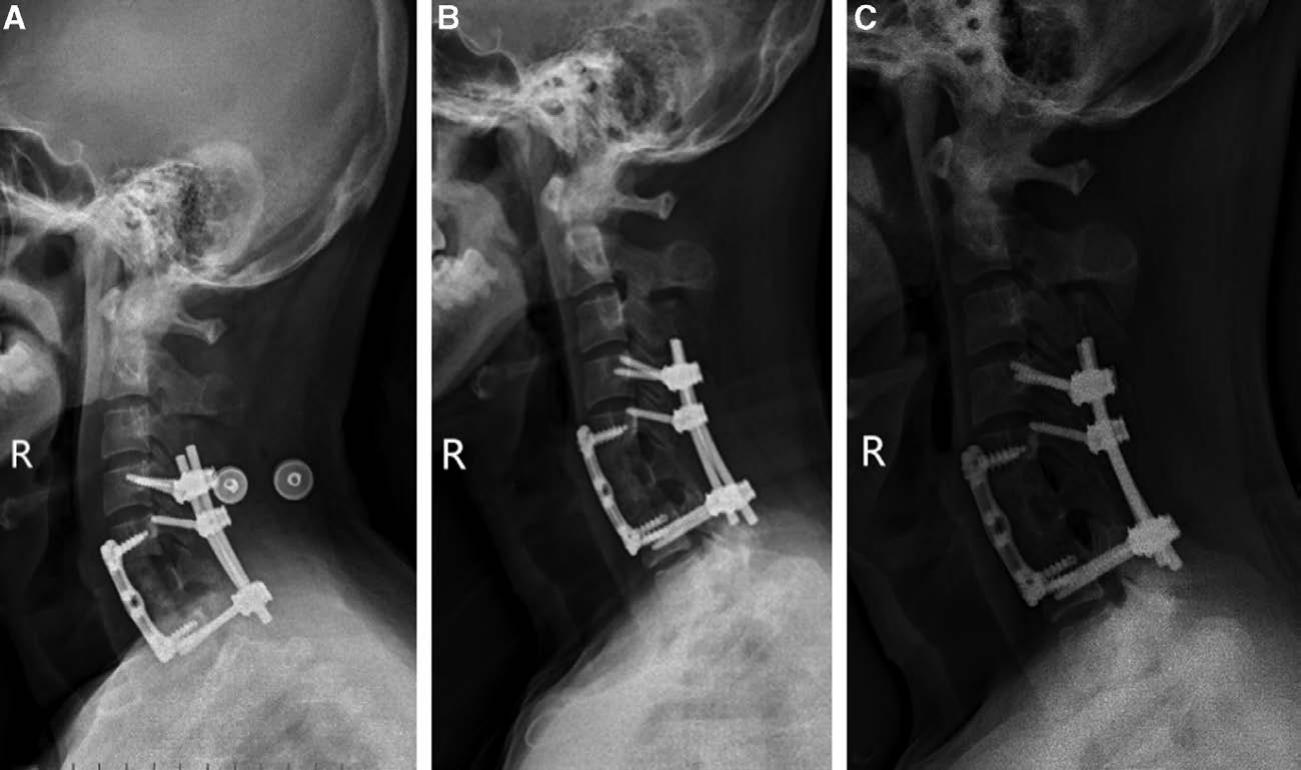
The postoperative (A) lateral X-ray image demonstrates the posterior fixation of the spine, and 3-month (B) and 9-month follow-up (C) X-ray images show a good level of calcification and filling of the lesion.

A biopsy from the C6 paravertebral soft tissue mass produced irregular red-brown fragments which were examined microscopically. It showed reactive fibroblastic tissue and increased osteoclastic activity with an accumulation of multinucleated giant cells, which was consistent with osteoclastoma (Fig. 3A). A mistaken diagnosis of osteoclastoma at C6 was made. Following the images, laminectomy of C5-6 was performed. The tumor was euangiotic and fragile, and infiltrated muscle and vertebra. The tumor was removed as much as possible. The cervical spine was stabilized with bone screws, which were placed in the lateral mass of C4, the right lateral mass of C5, and the pedicles of C7. Because the lesions involved the vertebral body, the patient underwent anterior corpectomy and decompressive surgery in which vertebral body reconstruction with artificial bone and fusion with a plate (Fig. 4A). No pressure was noted on the spinal cord at the conclusion of the operation. His pain subsided completely, and the neurological deficit was evidently improved in the early postoperative period.

**Figure 5.**
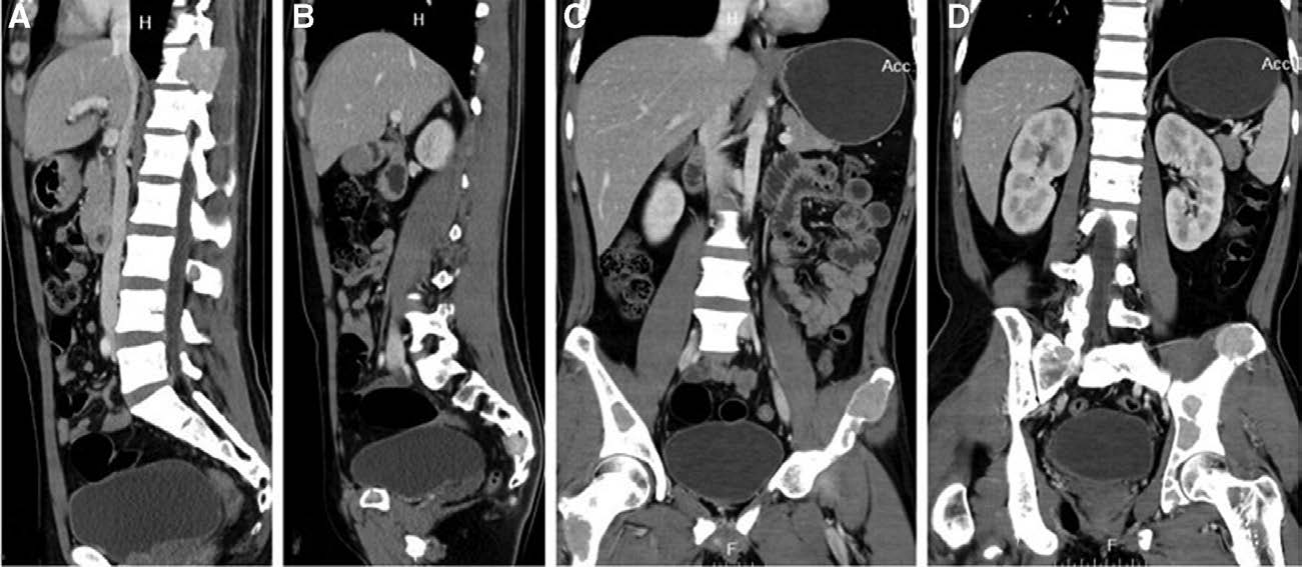
(A-D) CT images reveal multiple osteolytic lesions of the eleventh thoracic vertebrae, sacrum, pelvis, and femur. CT = computed tomography.

**Figure 6.**
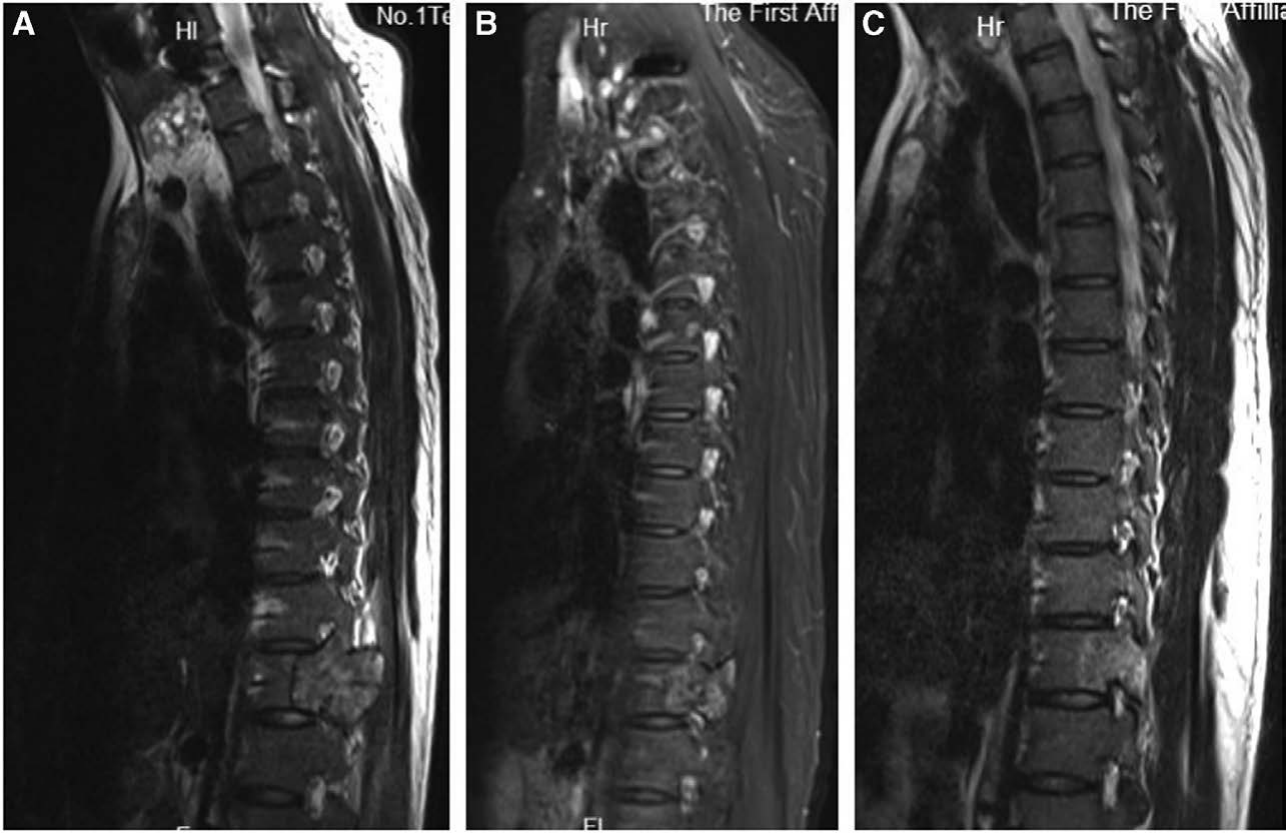
Compared with the first MRI (A) at the eleventh thoracic vertebrae, 3-month (B) and 9-month follow-up (C) MRI images show a significantly decrescent lesion. MRI = magnetic resonance imaging.

**Figure 7.**
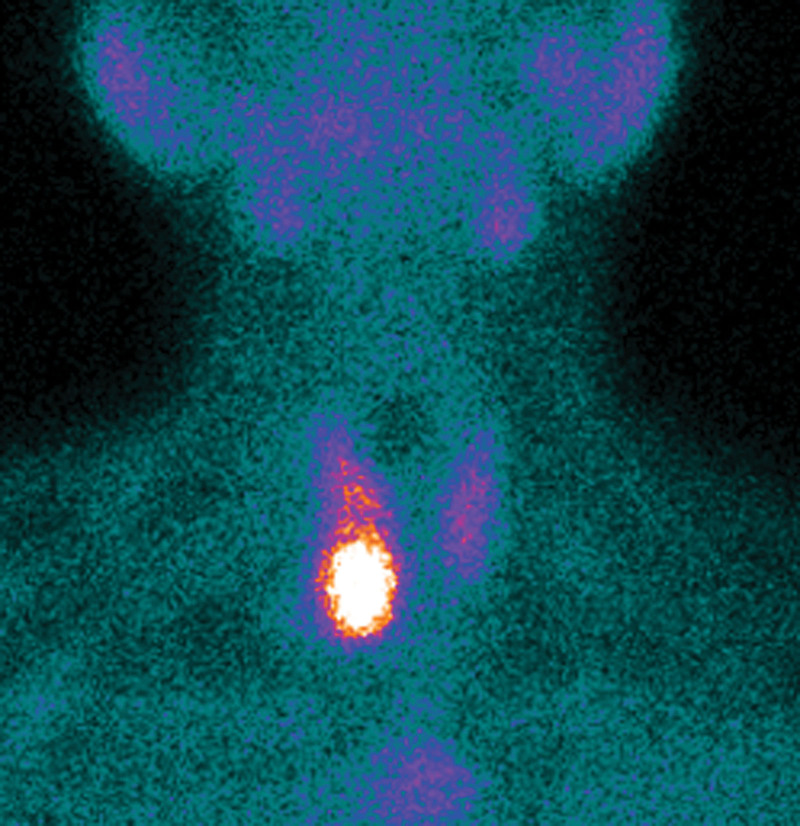
SPECT-CT image shows “a hot area” in the lower right parathyroid. SPECT-CT = single-photon emission computed tomography/computed tomography.

One month later, the patient was re-hospitalized with nausea and vomiting. Over this period, he had lost 5 kg in weight. Abdominal CT (Fig. 5) and MRI (Fig. 6A) showed a right renal calculus and multiple osteolytic lesions of the pelvis, femur, eleventh thoracic vertebrae, and sacrum. The fasting serum calcium varied between 2.82 and 3.76 mmol/L, and the PTH level elevated to 1438 pg/mL (normal 15–65 pg/mL). The diagnosis of primary hyperparathyroidism with BT due to a parathyroid tumor was considered. SPECT-CT of the parathyroid showed “a hot area” in the region of the lower right parathyroid (Fig. 7). The right parathyroid gland was surgically resected, and a microscopic examination was performed. Histology of the parathyroid showed an adenoma consisting mostly of chief cell type with no evidence of malignancy (Fig. 3B).

On the postoperative day 1, serum calcium and PTH level decreased to 2.66 mmol/L and 8.48 pg/mL, respectively. The patient had an uneventful postoperative course. Three-month (Fig. 4B) and 9-month follow-up X-ray (Fig. 4C) and CT scan showed a good level of calcification and filling of the lesion and MRI revealed that other lesions were reduced, and no recurrence occurred (Fig. 6B and C).

## 3. Discussion

Hyperparathyroidism is a clinical disorder in which the serum parathyroid hormone is increased.^[8]^ The increasing parathyroid hormone level will improve osteoclastic cell activity which can cause a series of changes, such as the decreasing of bone trabeculae, the proliferation of fibrous tissue, hemorrhage, and deposition of hemosiderin. These changes give the surrounding stroma a brown color, which is the name BT comes from. BT is found in <5%^[2]^ of patients with primary hyperparathyroidism and the incidence in secondary hyperparathyroidism is 1.55% to 13%^[3,4]^ Primary hyperparathyroidism is protean in its manifestations, and sometimes it is asymptomatic.^[9]^ These make the diagnosis of it very difficult and increase the difficulty to diagnose BT. Diagnosis of BT depends on clinical manifestation, pathological examination, image data, and biochemical tests.^[3]^

In this case, the patient was misdiagnosed with osteoclastoma. Osteoclastoma and BTs can both present pain and compression symptoms due to increased pressure. Furthermore, bone cystic changes can be found in CT in BTs, sometimes presented as swelling changes. When the lesion is multilocular, it is difficult to differentiate it from giant cell tumors of bone such as osteoclastoma and aneurysmal bone cysts. And the histopathologic results of the 2 tumors above are also highly similar, the tumor body of BTs contains giant bone cells, spindle stromal cells, and hemosiderin granules, accompanied by fibrous tissue hyperplasia and degeneration, which is also similar to other giant cell diseases, especially osteoclastoma. The vital point is, in this case, the importance of hypercalcemia found in the initial exam was ignored. When hypercalcemia was found, PTH may be needed and BT should be excluded to make the diagnosis accurate. Without the PTH level, misdiagnosis would appear.

To review this uncommon disease, we searched PubMed and Embase databases for similar case reports published since January 2022. The keywords were as follows: “Hyperparathyroidism,” “Brown tumor,” “Spine,” “Cervical,” “lumbar,” “vertebral,” and “thoracic.” A total of 51 patients with BTs were included, the detailed information on patients with primary hyperparathyroidism is shown in Table 1, while those with secondary hyperparathyroidism are shown in Table 2. Nerve compression symptoms may be the initial clinical manifestation while BT involves the spine in the setting of primary hyperparathyroidism. As shown in Tables 1 and 2, paraparesis (66.7%, 15 of 23 patients in primary hyperparathyroidism and 19 of 28 patients in secondary hyperparathyroidism) and pain (66.7%, 14 of 23 patients in primary hyperparathyroidism and 20 of 28 patients in secondary hyperparathyroidism) were the most seen symptoms due to the spinal cord compression, followed by radicular pain (35.3%, 13 of 23 patients in primary hyperparathyroidism and 7 of 28 patients in secondary hyperparathyroidism). Uroclepsia,^[10]^ dysuria,^[1]^ urinary retention,^[9]^ and sphincter dysfunction^[11]^ were other presenting symptoms of the patients. In this case, the patient manifested neck pain and radicular pain in both upper limbs.

**Table 1:** Literature review of spinal Brown tumors seen in primary hyperparathyroidism.

Author (yr)	Age	Sex	Affected spinal level	Symptoms	Treatment	Calcium (normal)	PTH (normal)
Shaw et al (1968)^[17]^	58	F	T10 pedicle	back pain paraparesis urinary retention	Tumor resection Parathyroidectomy Vitamin D and calcium	14.8 mg/dL (9–11)	Not reported
Shuangshoti et al (1972)^[18]^	32	M	L4 posterior elements	radicular pain paraparesis	Tumor resection Parathyroidectomy	Not reported	Not reported
Siu et al (1977)^[9]^	64	F	T10 multiple	back pain paraparesis urinary retention	Tumor resection Parathyroidectomy	14 mg/dL	15 ng/mL (0.7)
Ganesh et al (1981)^[19]^	40	M	T2 posterior elements	radicular pain paraparesis	Parathyroidectomy	3.65 mmol/l	Not reported
Yokota et al (1989)^[1]^	58	F	T5 posterior elements	back pain paraparesis dysuria	Tumor resection Parathyroidectomy	13.0 mg/dL (8.2–10.5)	1.8 ng/mL (<0.5)
Daras et al (1990)^[5]^	54	F	T9 pedicles multiple	back pain paraparesis	Tumor resection	13.7 mg/dL	Not reported
Kashkari et al (1990)^[12]^	51	F	T6-7	back pain paraparesis	Tumor resection Parathyroidectomy	13.7 mg/dL (8.4–10.6)	105 pg/mL (4–19)
Graziani et al(1991)^[20]^	64	F	C6	radicular pain paraparesis	Tumor resection Parathyroidectomy	Not reported	Not reported
Sarda et al (1993)^[21]^	23	F	T3-4	radicular pain paraparesis	Tumor resection Parathyroidectomy	10.8 mg/dL (9–11)	Not reported
Motateanu et al (1994)^[22]^	57	M	L4-5 posterior elements multiple	radicular pain	Tumor resection	2.78 mmol/L (2.25–2.75)	0.37 ng/mL (<0.28)
Ashebu et al (2002)^[23]^	27	F	C6 multiple	paraparesis	Parathyroidectomy Calcium, magnesium and vitamin D	3.4 mmol/L (2.1–2.8)	67 pmol/L (1.3–7.7)
Mustonen et al (2004)^[24]^	28	M	L2 posterior elements	back pain radicular pain	Parathyroidectomy	3.65 mmol/L (2.2–2.5)	930 ng/L (12–72)
Haddad et al (2007)^[25]^	62	F	T2-3 posterior elements multiple	paraparesis	Tumor resection Parathyroidectomy Vitamin D and calcium	12.4 mg/dL (8.5–10.2)	>1000 pg/mL (25–52)
Altan et al (2007)^[26]^	44	F	S2	back pain	Tumor resection Parathyroidectomy Calcium	11.40 mg/dL (8.5–10.5)	87.40 pg/mL (10–65)
Khalil et al (2007)^[27]^	69	M	L2 multiple	radicular pain	Tumor resection	Not reported	Not reported
Hoshi et al (2008)^[28]^	23	F	Sacrum multiple	back pain radicular pain	Parathyroidectomy	12.9 mg/dL (8.7–10.1)	3200 pg/mL (160–520)
Alfawareh et al(2013)^[2]^	26	F	C2 multiple	neck pain radicular pain	Parathyroidectomy	3.45 mmol/L (2.0–2.52)	64.4 pmol/L (1.6–6.9)
Lee et al (2013)^[29]^	65	M	L2 body L1 spinous multiple	back pain radicular pain	Tumor resection Parathyroidectomy Vitamin D and calcium	12.8 mg/dL (8.2–10.5)	1,889 pg/dL (0–65)
Khalatbari et al (2014)^[30]^	16	M	L2 posterior elements	back pain radicular pain paraparesis sphincter dysfunction	Tumor resection Parathyroidectomy	11 mg/dL (8.5–10.).	1597 pg/mL (12–70)
Khalatbari et al (2014)^[30]^	46	F	L3 posterior elements	back pain paraparesis radicular pain	Tumor resection Parathyroidectomy	Not reported	2060 pg/mL (12–70)
Khalatbari et al (2014)^[30]^	52	F	C6 posterior elements	neck pain radicular pain	Tumor resection Parathyroidectomy	Not reported	865 pg/mL (12–70)
Khalatbari et al (2014)^[30]^	38	M	T7 body and posterior elements	paraparesis sphincter dysfunction	Tumor resection	Not reported	Normal
Sonmez et al (2015)^[31]^	50	M	T9 posterior elements	back pain paraparesis sphincter dysfunction	Tumor resection Parathyroidectomy	14.3 mg/dL (8.4–10.2)	547.45 pg/mL (15–68.3)
Current case	29	M	C6 body and posterior elements multiple	neck pain radicular pain	Tumor resection Parathyroidectomy	3.51 mmol/L (2–2.7)	1438 pg/mL (15–65)

**Table 2:** Literature review of spinal Brown tumors seen in secondary hyperparathyroidism.

Author (yr)	Age	Sex	Affected spinal level	Symptoms	Treatment
Ericsson et al (1978)^[32]^	47	F	C7-T1	interscapular pain paraparesis	Tumor resection Parathyroidectomy
Bohlman et al (1986)^[16]^	69	F	T8 pedicle multiple	paraparesis	Tumor resection Steroids
Pumar et al (1990)^[33]^	24	F	T8 posterior elements multiple	paraparesis	Tumor resection
Barlow et al (1993)^[34]^	31	F	C5	neck pain radicular pain	Orthosis Parathyroidectomy
Kharrat et al (1997)^[35]^	46	M	Thoracic and Lumbar multiple	radicular pain	Parathyroidectomy
Mourelatus et al (1998)^[10]^	48	M	T2 body and posterior elements multiple	paraparesis uroclepsia	—
Fineman et al (1999)^[36]^	37	F	T7-9 multiple	thoracic pain paraparesis	Tumor resection Parathyroidectomy
Azria et al (2000)^[37]^	40	F	Thoracic	back pain	Parathyroidectomy
Masutani et al (2001)^[38]^	39	F	T4 posterior elements T7	paraparesis	Tumor resection Parathyroidectomy
Torres et al (2001)^[39]^	24	F	T9 posterior elements	back pain paraparesis	Tumor resection Parathyroidectomy Vitamin D and calcium
Paderni et al (2003)^[40]^	45	F	L3 body and pedicle L2, L5, and S1	paraparesis	Tumor resection Parathyroidectomy
Vandenbussche et al (2004)^[41]^	37	F	T8 body and pedicle	back pain paraparesis	Tumor resection Parathyroidectomy Vitamin D and calcium
Tarrass et al (2006)^[42]^	42	M	S1	back pain radicular pain	Tumor resection Parathyroidectomy
Jackson et al (2007)^[4]^	29	F	L4 body and posterior elements C6–T2 posterior elements multiple	back pain radicular pain	Tumor resection Parathyroidectomy
Kaya et al (2007)^[43]^	72	M	T1 body and pedicle	brachialgia	Tumor resection
Ren et al (2008)^[44]^	47	M	T4 body and pedicle	paraparesis	Tumor resection Calcium
Mak et al (2009)^[14]^	65	F	T8 lamina	back pain paraparesis	Tumor resection
Zaheer et al (2009)^[45]^	30	M	T12 posterior elements	back pain paraparesis dysuria	Tumor resection
Pavlovic et al (2009)^[46]^	40	M	T9 body and pedicle multiple	back pain paraparesis	Tumor resection
Mateo et al (2010)^[47]^	34	F	C2 multiple	neck pain	Orthosis Parathyroidectomy
Gheith et al (2010)^[48]^	19	M	L3 body and posterior elements	back pain paraparesis	Tumor resection Parathyroidectomy Vitamin D and calcium
Gheith et al (2010)^[48]^	25	F	C4-5 body and posterior elements	neck pain radicular pain paraparesis	Tumor resection Parathyroidectomy Vitamin D and calcium
Resic et al (2011)^[49]^	27	M	C6 posterior elements	neck pain radicular pain	Tumor resection Parathyroidectomy
Bertal et al (2011)^[50]^	49	M	L1	back pain paraparesis	Tumor resection Parathyroidectomy
Duval-Sabatier et al (2011)^[51]^	32	M	T5	back pain	Tumor resection Parathyroidectomy
Fargen et al (2013)^[13]^	33	F	L1 body and posterior elements	back pain paraparesis	Tumor resection
Arujau et al (2013)^[15]^	47	M	T5 posterior elements sacrum	back pain paraparesis	Percutaneous ethanol injection therapy Tumor resection
Tayfun et al 2014^[3]^	26	F	T8	back pain paraparesis	Tumor resection

According to the analysis of the literature review and our case, the histopathological characteristics of BT are: numerous multinucleated osteoclastic giant cells, increased osteoclastic activity, bone trabeculae were reduced in number, and the remaining ones appeared thinned, proliferating fibrous tissue, and the vascular stroma, hemorrhage, and hemosiderin deposition. These characteristics are very similar histologically to other giant cell lesions, such as true giant cell tumors, reparative giant cell granuloma, and aneurysmal bone cysts. In this situation, only the clinical manifestation, endocrine status, and laboratory test results differentiate BT from other giant cell lesions.

On CT imaging, BTs appear as hyperdensity well-demarcated expansile lytic lesions with various amounts of bone destruction. The bone cortex may be destroyed and thinned. The tumor is rich in vascularity and can be strongly enhanced in the enhanced computed tomographic scan. The MRI appearance of BT is described as iso- or hypointense on T1 weighted images and hyper- or hypointense on T2 weighted images.^[2]^ The tumor can be intensely enhanced after contrast injection. In our case, the lesion was occupying the C6 body and left-posterior elements of C5/6 with cortical destroying and encasing the left vertebral artery on CT and MRI. It showed hypointense signals on T2-weighted images and isointense on T1-weighted images.

Clinical manifestation, pathological findings, and imaging characteristics of BT are generally nonspecific. It can imitate many other entities such as multiple myeloma, metastases, sarcomas, and other giant cell lesions.^[12]^ However, signs of hyperparathyroidism can be accurately found by the image.^[13]^ BTs in patients with secondary hyperparathyroidism are mostly caused by chronic renal failure. In this situation, only the endocrine level can differentiate BTs in patients with primary hyperparathyroidism from other giant cell lesions. In our review, 23 patients have spinal BT in the setting of primary hyperparathyroidism. The serum calcium level of 17 patients has been found and 16 of them (94.1%) have hypercalcemia. Meanwhile, the serum parathyroid hormone of 15 patients has been collected and only 1 of them (6.7%) was normal. So, we can come to the conclusion that endocrine level can be an indicator to differentiate BT in patients with primary hyperparathyroidism from other giant cell lesions. In this patient, the pathological diagnosis was consistent with osteoclastoma, and we ignored his serum calcium, which resulted in a mistaken diagnosis.

For causes of the formation of BT, the primary treatment is the management of underlying medical disorders caused by hyperparathyroidism. The strategies for primary and secondary hyperparathyroidism are different. Parathyroidectomy is the gold standard treatment for BT with primary hyperparathyroidism. For secondary hyperparathyroidism, monitoring and preventing it with prolonged dialysis sessions is the best treatment.^[14]^ If the tumor involves the spine, we should take different management strategies. BT of the spine that causes nerve compression symptoms may require emergency surgical management. Treatments of 50 patients were collected. 41 of 50 patients (82%, 18 of 23 patients in primary hyperparathyroidism, and 23 of 27 patients in secondary hyperparathyroidism) in our review underwent tumor resection and parathyroidectomy was performed in 36 of 50 patients (72%, 19 of 23 patients in primary hyperparathyroidism and 17 of 27 patients in secondary hyperparathyroidism). One patient was treated with percutaneous ethanol injection therapy^[15]^ and a patient did not accept the treatment of parathyroidectomy.^[5]^ After surgery, improvement of symptoms was observed in most patients and a patient died of numerous medical complications.^[16]^

Our search in the literature demonstrated 51 spinal BT patients with primary (Table 1) or secondary hyperparathyroidism (Table 2). In summary, 30 of 51 patients (59%) were women. The patients’ age ranged from 16 to 72 with a mean of 42.2 years. The thoracic spine was the most affected part of the spine (60.7%) followed by multiple (41.2%), lumbar (23.5%), cervical (17.6%), and sacral (9.8%) regions.

## 4. Conclusion

In summary, the present report emphasized that in patients presenting with a vertebral lesion, BT should be considered in the differential diagnosis, especially when giant cell tumors are considered, serum calcium and PTH exam may be needed to exclude BTs. Tumor resection is required while BTs of the spine cause neurological symptoms.

## Author contributions

**Conceptualization:** Zirui Liu, Liang Zhao.

Data curation: Zirui Liu.

Investigation: Zirui Liu, Hao Yang, Ruipeng Song, Yang Zhang.

Resources: Zirui Liu, Hongyu Tan.

Writing – original draft: Zirui Liu.

Writing – review & editing: Zirui Liu.
